# The nano‐windmill exerts superior anti‐inflammatory effects via reducing choline uptake to inhibit macrophage activation

**DOI:** 10.1111/cpr.13470

**Published:** 2023-04-13

**Authors:** Nanxin Liu, Yuke Zhong, Xiaoxiao Pang, Mingzheng Li, Richard D. Cannon, Li Mei, Xiaoxiao Cai, Ping Ji

**Affiliations:** ^1^ Stomatological Hospital of Chongqing Medical University, Chongqing Key Laboratory of Oral Diseases and Biomedical Sciences, Chongqing Municipal Key Laboratory of Oral Biomedical Engineering of Higher Education Chongqing P. R. China; ^2^ Department of Oral Sciences Sir John Walsh Research Institute, Faculty of Dentistry, University of Otago Dunedin New Zealand; ^3^ State Key Laboratory of Oral Diseases, National Clinical Research Center for Oral Diseases, West China Hospital of Stomatology Sichuan University Chengdu P. R. China

## Abstract

Macrophages' activation plays a central role during the development and progression of inflammation, while the regulation of metabolic reprogramming of macrophages has been recently identified as a novel strategy for anti‐inflammatory therapies. Our previous studies have found that tetrahedral framework nucleic acid (tFNA) plays a mild anti‐inflammatory effect by inhibiting macrophage activation, but the specific mechanism remains unclear. Here, by metabolomics and RNA sequencing, choline uptake is identified to be significantly repressed by decreased *slc44a1* expression in tFNA‐treated activated macrophages. Inspired by this result, combined with the excellent delivery capacities of tFNA, siR‐slc44a1 is loaded into the tFNA to develop a new tFNA‐based small interfering RNA (siRNA) delivery system named ‘nano‐windmill,’ which exhibits a synergetic role by targeting *slc44a1*, finally blowing up the anti‐inflammatory effects of tFNA to inhibit macrophages activation via reducing choline uptake. By confirming its anti‐inflammatory effects in chronic (periodontitis) and acute (sepsis) inflammatory disease, the tFNA‐based nanomedicine developed for inflammatory diseases may provide broad prospects for tFNA upgrading and various biological applications such as anti‐inflammatory.

## Graphical abstract

A tFNA‐based nanomedicine named ‘nano‐windmill,’ which loads siR‐slc44a1 on tFNA through a sticky end containing RNase H‐responsive sequence, is developed to amplify the anti‐inflammatory effects of tFNA by synergistically decreasing *slc44a1* expression, thus reducing choline uptake, and finally inhibiting macrophages overactivation. This novel tFNA‐based siRNA delivery system paves the way for the development of new nanomedicine with superior anti‐inflammatory effects.

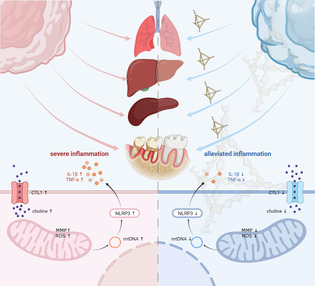

## 1 INTRODUCTION

Inflammatory diseases are usually caused by host immune responses induced by pathogenic microorganisms or physical and chemical factors.^1^, ^2^ As one of the most critical innate immunocytes, macrophages play essential roles in the progression of inflammatory diseases by affecting immune homeostasis.^3^, ^4^ Over‐activated macrophages release a large amount of pro‐inflammatory cytokines, which induce an inflammatory cascade reaction, aggravate the inflammatory response, and cause tissue inflammatory damage. Therefore, inhibiting macrophage overactivation has become an effective therapeutic strategy for inflammatory diseases.

Accumulated evidence suggests that cellular metabolism controls the phenotypic and functional changes of macrophages.^5^, ^6^ Thus, many researchers are committed to exploring the emerging new area of immune metabolism to find potential therapeutic targets for unwanted inflammation.^7^, ^8^ Recently, the metabolism of choline, an essential human nutrient required for synthesizing membrane phospholipids and acetylcholine, has been identified to be associated with macrophage activation.^9^, ^10^ Targeted reduction of slc44a1‐encoded choline‐transporter‐like protein 1 (CTL1) expression effectively restrained macrophage overactivation by inhibiting choline uptake,^9^ which suggests that reprogramming of choline metabolism may be a promising treatment for inflammatory diseases via restraining macrophage overactivation.

Tetrahedral framework nuclear acid (tFNA), assembled by four specific deoxyribonucleic acid (DNA) single chains (S1–S4), is a nanomaterial with great application potential in the biomedical field.^11^, ^12^, ^13^, ^14^ Our previous studies have found that tFNA could enter macrophages and exert mild anti‐inflammatory and antioxidant effects,^15^, ^16^, ^17^ while the specific mechanism of how tFNA repressed the overactivation of macrophages remains elusive. In this study, by metabolomics and RNA sequencing, we found that *slc44a1*‐mediated choline uptake may be the critical driving factor for tFNA inhibiting macrophage overactivation. Additionally, we previously identified that tFNA is an excellent delivery platform due to its superior cell and tissue permeability, good biocompatibility, and programmability.^18^, ^19^, ^20^, ^21^, ^22^, ^23^, ^24^, ^25^ Therefore, siR‐slc44a1 was loaded into the tFNA to create a new tFNA‐based siRNA delivery system, named ‘nano‐windmill,’ which could play a more potent anti‐inflammatory effect than tFNA by safely increasing the knockdown efficiency of *slc44a1*.

To our knowledge, this is the first study to explore the anti‐inflammatory mechanism of tFNA from the perspective of metabolism and apply a new siRNA delivery strategy to upgrade tFNA based on metabolic reprogramming. The nano‐windmill developed by combining the metabolomics results and the nano‐carrier characteristics of tFNA not only ensured the delivery and protection of siR‐slc44a1, but also synergistically targeted slc44a1 to reduce the choline uptake of macrophages, thus inhibiting macrophage activation, finally playing an excellent anti‐inflammatory effect in vitro and in vivo. Notably, through a specific assembled strategy, the tFNA in the ‘nano‐windmill’ maintains the intact structure even after releasing the siR‐slc44a1, which contributes to the more slc44a1 knockdown level and superior anti‐inflammatory effects. Together, our results provide a new tFNA upgrading strategy to amplify its biological effects and may pave the way to developing a novel anti‐inflammatory agent.

## 2 MATERIALS AND METHODS

### 2.1 Synthesis of tFNA and the nano‐windmill

The tFNA was synthesized as previously described.^26^ As to the nano‐windmill, four single DNA strands modified by adding sticky ends at the 5′ ends (sS1‐sS4) went through a mixed and annealing process to obtain stFNA with four sticky‐end apexes. Then, stFNA was incubated with siR‐slc44a1 at room temperature for 30 min. All sequences were listed in Table S2.

### 2.2 Characterization and cellular uptake of the nano‐windmill

8% PAGE and TEM were performed to confirm the successful synthesis of the nano‐windmill. Zeta potential and particle size of the nano‐windmill were measured by DLS. The tFNA, nano‐windmill, and tFNA‐NC synthesized using cyanine‐5 ssDNA single‐stranded DNA (Cy5)‐S1 or Cy5‐sS1 chain were incubated with cells for 3, 6, and 24 h, and then flow cytometry was performed. Then, Cy5 was connected to siR‐slc44a1. Cy5‐siR‐slc44a1 was transfected using lipofectamine 2000, Cy5‐nanowindmill was directly incubated with cells, and the entry of siR‐slc44a1 into cells was detected by confocal microscopy after 24 h.

### 2.3 Enzyme cleavage test and siRNA stability test

The nano‐windmill was incubated with RNase H (Sangon) at 0, 25, 50, 100, and 200 U/mL at 37°C for 1 h to observe its separation by PAGE. SiRNA and nano‐windmill were incubated with fetal bovine serum (FBS, 0%, 2.5%, 5%, 10%, 20%, 40%) for 30 min or 5% FBS for 0, 0.5, 1, 2, 4, and 8 h. Furthermore, siRNA and nano‐windmill were incubated with a gradient concentration of RNase A (0, 0.05, 0.1, 0.5, 1, 2 U/mL) for 30 min, or with RNase A (0.1 U/mL) for a gradient time (0, 0.5, 1, 2, 4, and 8 h) at 37°C. The stability of siRNA treated with FBS or RNase A was observed by agarose gel electrophoresis.

### 2.4 Cell culture and stimulation

This study was approved by the ethics committee of Stomatology Hospital Affiliated to Chongqing Medical University. The approval number is 2022‐110. All animal experiments met the NIH Guide for Care and Use of Laboratory Animals. Femurs and tibias from C57BL/6 mice at 6–9 weeks of age were obtained to generate BMDMs, as described in the previous study.^27^ Acquired BMDMs were treated with tFNA (250 nM) or the nano‐windmill for 6 h without FBS and penicillin–streptomycin. SiR‐slc44a1 was transfected for 6 h in vitro using Lipofectamine 2000 (Thermo Fisher) according to the manufacturer's instructions unless otherwise indicated. Next, BMDMs were first treated with ultrapure lipopolysaccharide (LPS, 100 ng/mL) for 4 h, followed by activator adenosine triphosphate (ATP) (4 mM) for 45 min. THP‐1 cells were cultured in RPMI‐1640 (Sigma) supplemented with 10% FBS and 1% penicillin–streptomycin and induced with PMA (100 ng/mL) for 24 h to differentiate into macrophages.

### 2.5 Quantitative real‐time PCR

Total RNA was extracted using RNAeasy™ Animal RNA isolation kit with the spin column, and complementary DNA (cDNA) was synthesized with PrimeScript™ RT Master Mix (Takara Bio, Shiga). The mRNA expression was determined by Bio‐Rad real‐time PCR system.

### 2.6 Western blot

Cells were washed with cold phosphate buffer saline (PBS) twice and lysed with RIPA lysis buffer containing protease and phosphatase inhibitor cocktail (Thermo Scientific™ Halt). After centrifugation of cell lysates, proteins in supernatants were separated by SDS‐PAGE, transferred to polyvinylidene difluoride (PVDF) membrane, blocked in 5% bovine serum albumin (BSA), and incubated with anti‐slc44a1 polyclonal antibody (1:1000, Proteintech), anti‐Caspase‐1 (1:1000, ab179515), anti‐NLRP3 (1:1000, ab263899), anti‐IL‐1β (1:1000, ab283818) overnight at 4°C. The secondary antibody (1:3000, Beyotime) was added to incubate with protein samples for 1 h, and detection was performed by the ECL chemiluminescence system (Bio‐Rad, Hercules).

### 2.7 Elisa

Mouse IL‐1β Valukine Elisa Kit (R&D Systems) was used to determine cytokine concentration according to the manufacturer's instructions. Briefly, prepare all reagents and working standards as indicated, then add standard or sample to a microplate and incubate for 2 h. Next, IL‐1β conjugate, substrate solution, and stop solution were added in turn. Finally, a microplate reader set to 450 nm and 540 nm was used to determine the optical density of each well.

### 2.8 Measurement of cytosolic mitochondrial DNA (mtDNA)

Cytosolic mtDNA was detected by qPCR, as described in previous studies.^28^, ^29^ Cells were lysed by 0.1% NP‐40 (Igepal CA‐630) solution and incubated on ice for 15 min. Cell lysates were centrifuged at 13,000 rpm for 15 min at 4°C. Supernatants were collected to extract cytosolic mtDNA using DNeasy Blood & Tissue Kit (Qiagen) according to the manufacturer's instructions. QPCR was employed to measure cytosolic mtDNA.

### 2.9 Analysis of reactive oxygen species (ROS) and mitochondrial membrane potential (MMP)

ROS was detected by ROS Assay Kit (Beyotime) according to the manufacturer's instructions. Briefly, cells were stained with 10 uM DCFH‐DA for 20 min at 37°C after treatment. Fluorescence intensity was measured by BMG. MMP was evaluated using an MMP assay kit with JC‐1 (Beyotime) according to the manufacturer's instructions.

### 2.10 Immunofluorescence

BMDMs were washed twice, fixed by 4% cold paraformaldehyde for 30 min, permeabilized by 0.5% Triton X‐100 for 10 min, blocked by goat serum for 1 h, and incubated with anti‐ATPB (1:100, Abcam) overnight at 4°C. On the second day, BMDMs were stained with Goat anti‐mouse IgG H&L (Alexa Flour® 488, Abcam) for 1 h, followed by a second block and primary incubation (anti‐dynamic‐related protein 1 (anti‐Drp1), 1:250, Abcam; anti‐SQSTM1/p62, 1 μg/mL, Abcam). On the third day, a secondary antibody (Alexa Flour® 555, Abcam) was added to incubate with cells for 1 h. DAPI was used to stain the nucleus. Slides and stained cells were observed and photographed by a confocal microscope.

### 2.11 LPS‐induced septic shock

C57BL/6 mice (male, eight‐ to ten‐week‐old) were randomly divided into five groups. Mice in four groups were induced septic shock by intraperitoneal injection (IP) injection of LPS (10 mg/kg), and mice in the control group were IP injected with equal volume saline. 200 μL of saline, tFNA, siR‐slc44a1, or the nano‐windmill were injected daily into mice in the four groups via the tail vein 2 days before the LPS challenge and on LPS injection day. After LPS stimulation for 24 h, all mice were euthanized to analyse the expression of inflammatory cytokines and injury of various organs. Mice were IP injected with 50 mg/kg LPS to analyse for survival.

### 2.12 Wet/dry (W/D) weight ratio

After cutting the air tube, the whole lung was taken and weighed by wet weight, and then the lung was dried in a 65°C incubator. After 24 h, the lung was removed and weighed by dry weight, and the wet/dry weight ratio of the lung was calculated.

### 2.13 Histological analysis

The lung, spleen, and liver were collected from mice that were sacrificed after 24 h of LPS treatment (10 mg/kg). These tissues were fixed, dehydrated, embedded in paraffin, sliced, and then subjected to haematoxylin and eosin (H&E) staining. Morphological changes were evaluated by a semi‐quantitative scoring system. The lung injury score is based on the presence of congestion, haemorrhage, infiltration of neutrophils, debris, and hyperplasia, each ranging from 0 to 3.^30^ The liver tissue was scored for hepatocyte degeneration, lobular inflammation, thrombus formation, and necrosis (each 0–3).^31^ The histological score of the spleen was evaluated according to necrosis and the enlargement of lymphocyte areas in red and white pulps, which were graded 0–2 and 0–3, respectively.^32^ The total score was represented as the sum of different scores for each parameter.

### 2.14 Ligature‐induced periodontitis and analysis

The bilateral maxillary second molars of eight‐to‐ten‐week‐old mice were ligated with 5–0 silk sutures for 2 weeks to induce periodontitis. During ligation, mice were treated with saline, siR‐slc44a1, tFNA, and nano‐windmill, respectively. Mice in the control group received no treatment. Two weeks later, all mice were sacrificed, and maxillae were taken for micro‐CT scanning. H&E staining was performed after demineralization of periodontal tissues.

### 2.15 Statistical analysis

All experiments were performed independently with similar results at least three times. Data were shown as mean ± SD. Data analysis was obtained from GraphPad Prism 9 software (GraphPad Software) through One‐way analysis of variance, two‐tailed Student's *t*‐test analysis, or Log‐rank (Mantel‐Cox) test.

## 3 RESULTS

### 3.1 Design, synthesis and characterization of the nano‐windmill

**FIGURE 1:**
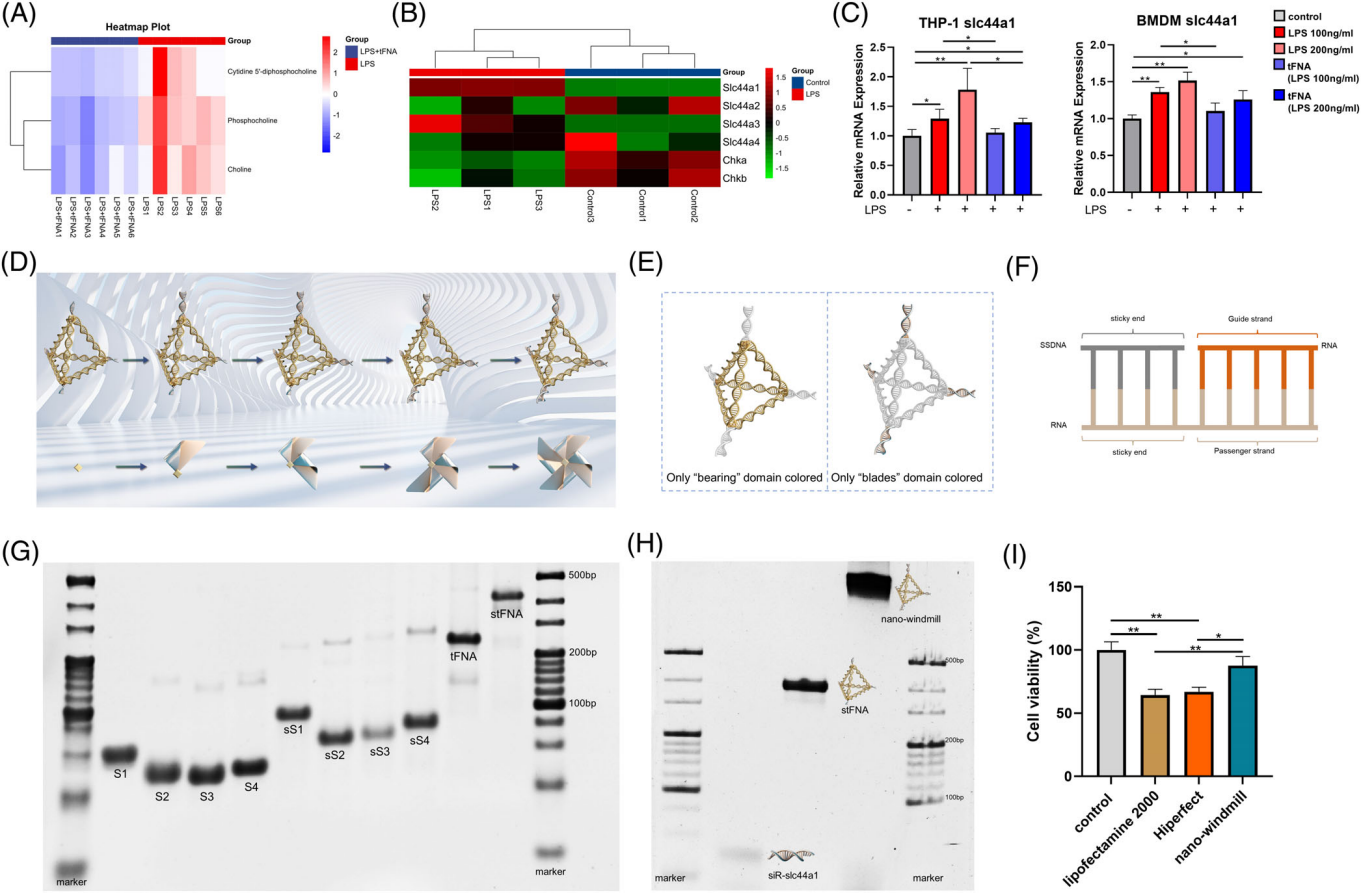
Design, synthesis and characterization of the nano‐windmill. (A) Relative content of metabolites analysed by untargeted metabolomics using LC–MS/MS, *n* = 6. (B) The heat map of mRNA transcription levels in bone marrow‐derived macrophages (BMDMs), *n* = 3. (C) Relative mRNA expression of slc44a1 in BMDMs and phorbol myristate acetate (PMA)‐differentiated THP‐1 cells treated as indicated, *n* = 3. (D) Schematic showing the fabrication of the nano‐windmill. (E) Schematic diagram of the ‘bearing’ and ‘blades’ domain. (F) Schematic showing the connection between stFNA and siR‐slc44a1. (G) An image of 8% PAGE confirming the synthesis of the ‘bearing’ domain. (H) PAGE showing the successful synthesis of the nano‐windmill. (I) The biosafety of the nano‐windmill, lipofectamine 2000, and Hiperfect transfection reagent was evaluated by CCK‐8 assay. Mean ± standard deviation (SD), *n* = 3. *p < 0.05, **p < 0.01.

First, we confirmed that tFNA could slightly inhibit the expression of pro‐inflammatory cytokines in LPS‐treated THP‐1 cells (Figure S1). Then, an untargeted metabolic screen was taken to examine the metabolic profile of THP‐1 cells stimulated by LPS or tFNA and LPS, and the results showed that choline, phosphocholine, and cytidine 5′‐diphosphocholine were decreased in LPS‐stimulated THP‐1 cells treated by tFNA (Figures 1A and S2; Table S1). Meanwhile, RNA sequencing revealed that *slc44a1*, the encoding gene of CTL1, which is responsible for choline uptake, was significantly upregulated by LPS but decreased after tFNA treatment (Figure 1B,C). Collectively, these results strongly indicated that tFNA could inhibit LPS induced‐macrophage activation by repressing choline uptake via decreasing *slc44a1* expression.

To amplify the ability of tFNA to repress macrophage overactivation, we designed a new tFNA‐based siRNA delivery system by connecting the ‘blades’ domain of the nano‐windmill to the ‘bearing’ domain via RNase H‐responsive sequence in the sticky end (Figures 1D–F and S3A), which would keep the intact structure of tFNA after siRNA releasing, finally leading to a more knockdown level of the targeted gene. Since siR‐slc44a1 is loaded on the four vertices of tFNA containing sticky ends (stFNA), which looks like a windmill, it is named as ‘nano‐windmill’ (Figure 1D). The polyacrylamide gel electrophoresis (PAGE) gel showed that the sticky end successfully modified the 5′‐end of each single DNA strand to synthesize stFNA (Figure 1G), and then siRNA duplex could be linked to stFNA (Figure 1H). Moreover, the transmission electron microscopy (TEM), dynamic light scattering (DLS) and CCK‐8 results showed that the nano‐windmill is a nanoparticle with low cytotoxicity, a size of about 34.07 ± 1.8 nm, a negative charge, and a tetrahedral structure (Figures 1I and S3B).

### 3.2 Effect of the nano‐windmill for delivery, protection, and release of siR‐slc44a1

**FIGURE 2:**
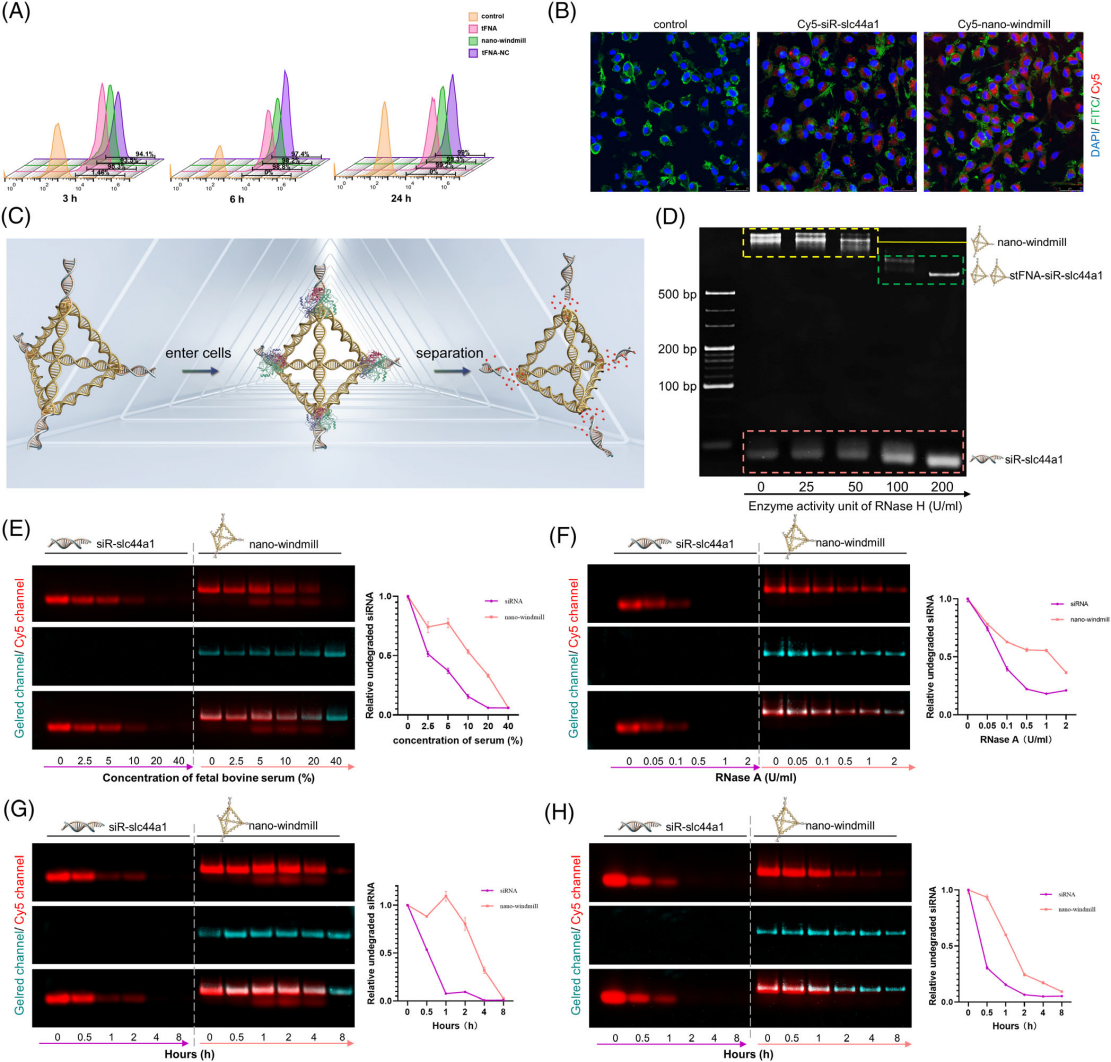
The nano‐windmill could deliver, protect and release siR‐slc44a1. (A) Flow cytometric results for cellular uptake of the nano‐windmill, tFNA, and tFNA‐negative control(tFNA‐NC), which were synthesized with Cy5‐sS1or Cy5‐S1. (B) Confocal images displaying the cellular uptake of Cy5‐siR‐slc44a1 and Cy5‐nano‐windmill. Scale bar: 25 μm. (C) Schematic diagram of siRNA release in cells. (D) PAGE showed results of enzyme cleavage test of the nano‐windmill. (E, F) The agarose gel electrophoresis results showing the degradation of free siR‐slc44a1 and siR‐slc44a1 in the nano‐windmill after incubation with FBS or RNase A at a gradient concentration for 30 min. (G) The degradation of free siR‐slc44a1 and siR‐slc44a1 in nano‐windmill after incubation with 5% FBS for different durations was evaluated by agarose gel electrophoresis. (H) The degradation of free siR‐slc44a1 and siR‐slc44a1 in nano‐windmill after incubation with 0.1 U/mL RNase A for different durations was evaluated by agarose gel electrophoresis.

Since the nano‐windmill was developed to amplify the anti‐inflammatory effect by meeting the requirements including siRNA delivery and protection, and maintenance of tFNA topology, several experiments were conducted. First, results of flow cytometry and immunofluorescence images showed that the nano‐windmill was taken up by bone marrow‐derived macrophages (BMDMs) in large quantities in a short time (Figure 2A,B), which revealed its effectively delivered capacities. Second, the RNase H‐responsive sequence was designed to be degraded after entering the cell (Figure 2C), while siR‐slc44a1 gradually dissociated from the nano‐windmill accompanied by the increase of RNase H concentration, identifying the successful separation of the nano‐windmill into tFNA and siR‐slc44a1 (Figure 2D), which is different from the previous tFNA‐based delivery system that tFNA released siRNA with breaking itself and loosing its intact structure after entry into cells,^33^, ^34^ such as the nanobox‐siR system^35^ or modification of the S2 chain in tFNA.^36^ Third, Cy5 was linked to the siR‐slc44a1 for comparing the stability of siR‐slc44a1 in the nano‐windmill system and siR‐slc44a1 alone by observing the degradation of siR‐slc44a1. The results showed that at the same treatment time, with the increase of FBS or RNase A concentration, the fluorescence quenching rate in the nano‐windmill was slower than that in free Cy5‐siR‐slc44a1 (Figure 2E,F), and the degradation rate of siR‐slc44a1 in the nano‐windmill was much lower by 5% FBS or 0.1 U/mL RNase A treatment (Figure 2G,H), which indicated strong stability of siR‐slc44a1 in the nano‐windmill. Together, the above results strongly indicate that the nano‐windmill could efficiently deliver, protect and release siR‐slc44a1 into cells without destroying the structure of tFNA.

### 3.3 The nano‐windmill restrained macrophage activation by impairment of choline uptake in vitro

**FIGURE 3:**
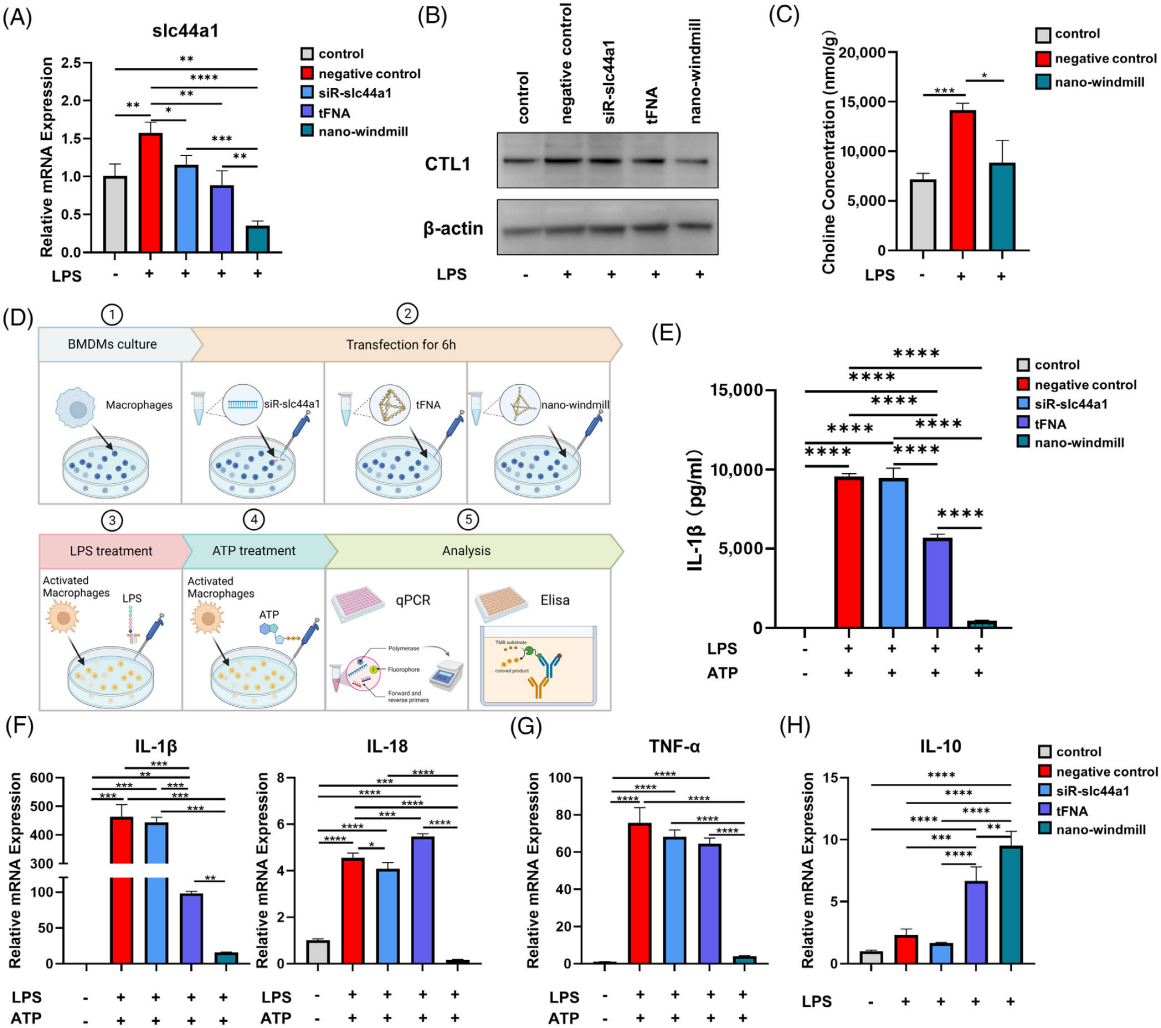
The nano‐windmill inhibited macrophage overactivation by targeting slc44a1 expression and decreasing choline uptake. (A) QPCR analysis of slc44a1 mRNA in BMDMs treated as indicated. Mean ± SD (*n* = 3). (B) Western blot analysis of CTL1. (C) Quantitative analysis of intracellular choline by targeted metabolomics. Mean ± SD (*n* = 3). (D) Schematic diagram of BMDM treatment. (E) Elisa analysis of IL‐1β release by BMDMs. Mean ± SD (*n* = 3). (F) The mRNA expression of IL‐1β and IL‐18 measured by qPCR. Mean ± SD (*n* = 3). (G, H) QPCR analysis of TNF‐α and IL‐10 mRNA expression, respectively. Mean ± SD (*n* = 3). *p < 0.05, **p < 0.01, ***p < 0.001, ****p < 0.0001.

Since the nano‐windmill was designed to gain a much more *slc44a1* knockdown level, the expression of *slc44a1* and CTL1 was examined. As expected, the knockdown efficiency of the nano‐windmill on *slc44a1* was higher than that of tFNA and siR‐slc44a1 alone (Figure 3A), while the expression of CTL1 encoded by *slc44a1* was significantly reduced after treatment of the nano‐windmill, verifying that tFNA and siR‐slc44a1 collectively silenced CTL1 after intracellular dissociation of the nano‐windmill (Figure 3B). Moreover, intracellular choline in BMDMs under different treatment conditions was quantified by targeted metabolomics, and the result showed that choline uptake was enhanced after the LPS challenge but impaired after the nano‐windmill treatment, correlating with CTL1 expression (Figure 3C).

Next, we detected the level of activation in BMDMs with different treatment (Figure 3D). As shown in the qPCR and Elisa data, tFNA and siR‐slc44a1 slightly influenced the expression of IL‐1β and IL‐18, which are downstream effectors of the NLRP3 inflammasome pathway, whereas the nano‐windmill significantly reduced their expression (Figure 3E,F). Also, the nano‐windmill had similar inhibitory effects on the expression of *TNF‐α*, another typical pro‐inflammatory cytokine (Figure 3G). On the other hand, the transcription level of the anti‐inflammatory cytokine *IL‐10* was not affected by siR‐slc44a1, slightly increased by tFNA, and sharply increased by nano‐windmill (Figure 3H). Together, these results demonstrate that the nano‐windmill could multiply the anti‐inflammatory effects of tFNA by inhibiting macrophage overactivation.

### 3.4 The nano‐windmill stimulated mitophagy to inhibit NLRP3 inflammasome activation

**FIGURE 4:**
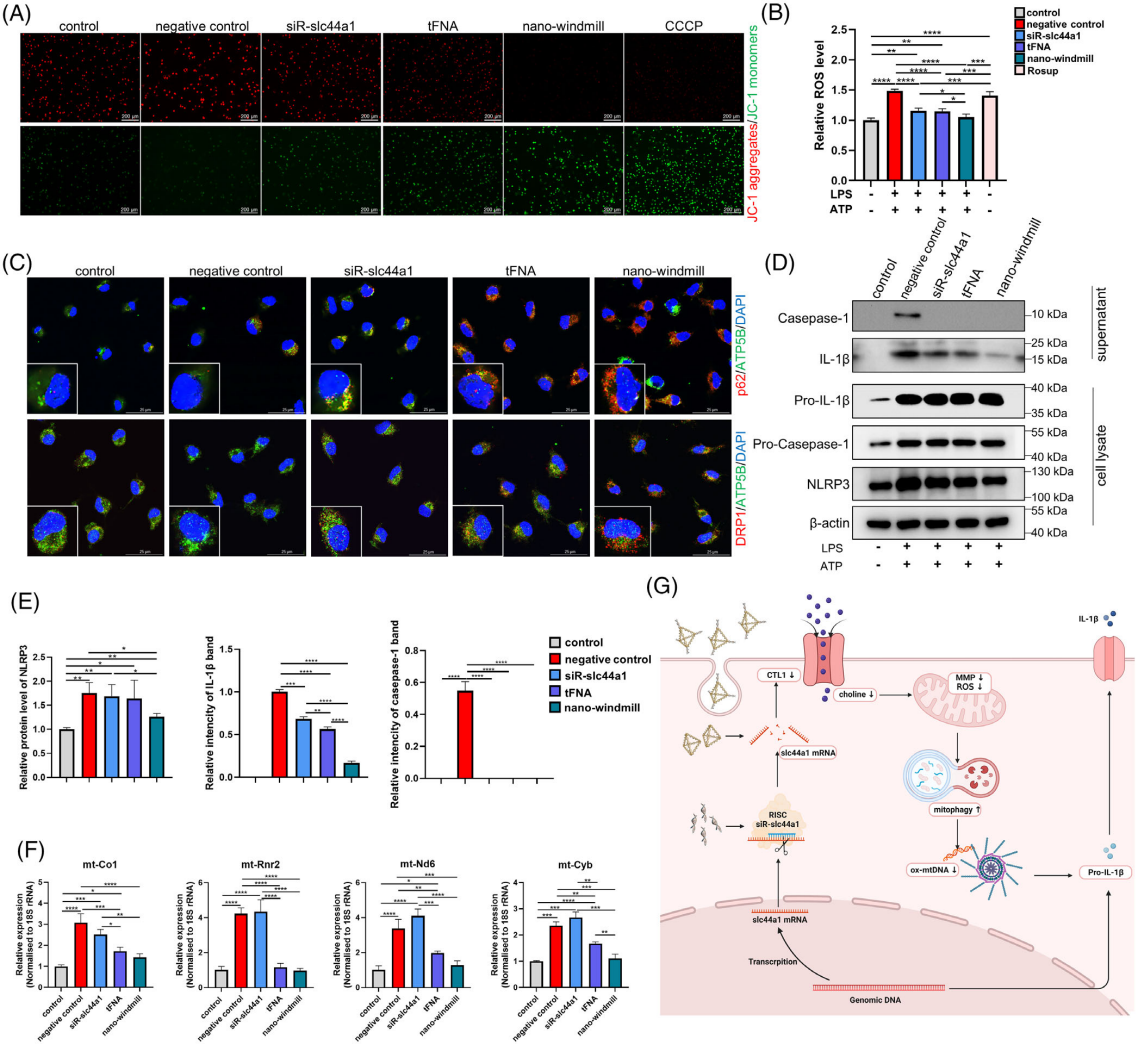
The nano‐windmill induced mitochondrial depolarization and mitophagy, resulting in repaired NLRP3 inflammasome activation. (A) Images stained with JC‐1 assay kit reflected MMP of BMDMs. (B) ROS production was measured in BMDMs treated as indicated. Mean ± SD (*n* = 4). (C) Immunofluorescence analysis of p62 and Drp1 recruitment to mitochondria. Mitochondria were stained green, nuclei blue, and p62 and Drp1 red. The scale bar represents 25 μm. (D) Western blotting results of indicated proteins in supernatants and cell lysates. (E) Histograms show the relative protein level of NLRP3, caspase‐1 p10, or mature IL‐1β. (F) Cytosolic translocation of mtDNA was quantified by qPCR. Cytosol fractions of BMDMs cultured and treated as indicated were analysed. Data in (D) and (G) were represented as mean ± SD, *n* = 3. *p < 0.05, **p < 0.01, ***p < 0.001, ****p < 0.0001. (G) Schematic diagram showing the anti‐inflammatory mechanism of the nano‐windmill.

Since the increase in mitochondrial membrane potential (MMP) and reactive oxygen species (ROS) promotes the pro‐inflammatory effects of LPS in macrophages, finally leading to macrophage activation,^37^ as we observed the elevated level of MMP and ROS in the activated THP‐1 cells (Figure S1), MMP and ROS were detected in the nano‐windmill treated BMDMs. The JC‐1 assay showed that green fluorescence accumulated in BMDMs treated by the nano‐windmill, indicating MMP of BMDMs was decreased by the nano‐windmill at least as effectively as carbonyl cyanide 3‐chlorophenylhydrazone (CCCP) (Figure 4A). Moreover, the nano‐windmill reduced ROS production, which was stronger than tFNA and siR‐slc44a1 (Figure 4B). Previous studies have found that decreased MMP, which represents mitochondrial depolarization, as a crucial factor in mitophage induction.^38^ We detected the mitochondrial recruitment of the autophagy adaper, and found that the nano‐windmill potentiated recruitment of p62/Sqstm1 and Drp1 to mitochondria, suggesting that the nano‐windmill may activate mitophagy which is responsible for reducing the production of ROS and cytosolic release of mtDNA through timely elimination of damaged mitochondria (Figure 4C).^39^, ^40^ As expected, the nano‐windmill did decrease the cytosolic release of mtDNA, and the cytosolic expression of mtDNA in the nano‐windmill group was lower than that in the tFNA group. However, siR‐slc44a1 seems to have no effect on this, which may be related to the transfection efficiency or the type of mtDNA tested (Figure 4F).

Next, the expression of NLRP3 inflammasome components was examined since cytosolic mtDNA and ROS production were reported to promote NLRP3 inflammasome formation and trigger inflammatory cytokine secretion.^41^, ^42^ According to data from the western blot, the nano‐windmill reduced the activation of caspase‐1 and NLRP3. Furthermore, diminished IL‐1β production detected in supernatant on the nano‐windmill treatment correlated with reduced caspase‐1 and NLRP3 activation (Figure 4D,E). Overall, the nano‐windmill was cleaved into tFNA and siR‐slc44a1 after entering BMDMs, which together target to silence slc44a1, inhibit the expression of CTL1 on the cytomembrane, reduce choline uptake, promote mitochondrial depolarization and mitophagy, decrease ROS production and mtDNA cytoplasmic release, thereby inhibiting NLRP3 inflammasome activation, and finally restraining the inflammatory cytokine secretion (Figure 4G).

### 3.5 The nano‐windmill alleviated sepsis induced by LPS

**FIGURE 5:**
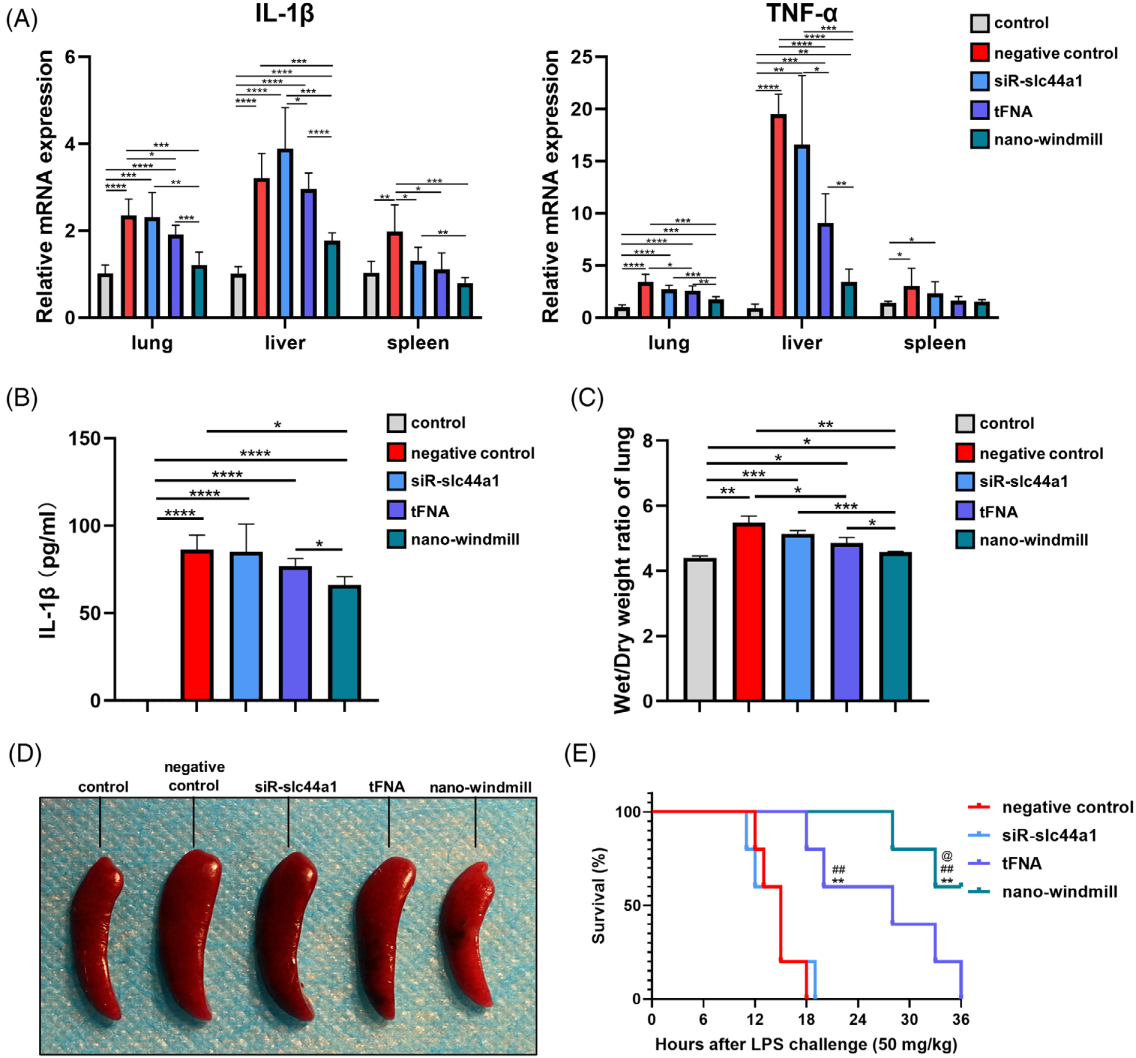
The nano‐windmill alleviated sepsis induced by LPS. (A) Statistical analysis of IL‐1β and TNF‐α mRNA expression in lung, liver, and spleen tissues of mice in different treatment groups. (B) Circulating IL‐1β was determined by ELISA. (C) W/D weight ratio reflecting lung tissue edema was recorded and statistically analysed. Data in (A–C) were represented as mean ± SD, *n* = 3. *p < 0.05, **p < 0.01, ***p < 0.001, ****p < 0.0001. (D) The image of spleens collected from different treatment groups. (E) % survival of mice was analysed by Kaplan–Meier analysis. *n* = 5 mice per group. *Compared with the negative control group, **p < 0.01; #Compared with the siR‐slc44a1 group, ##*p* < 0.01; @Compared with the tFNA group, @*p* < 0.05 (Log‐rank test).

To further validate the anti‐inflammatory effect and mechanism of the nano‐windmill in vivo, we established a sepsis model that is NLRP3 inflammasome dependent.^43^ At 24 h after intraperitoneal (IP) injection of 10 mg/kg LPS, lungs, livers, and spleens were harvested to assess the transcriptional level of pro‐inflammatory cytokines. Expression of *IL‐1β* and *TNF‐α* in lung, liver, and spleen tissue was sharply increased compared to mice in the control group. The nano‐windmill significantly attenuated the mRNA expression of *IL‐1β* and *TNF‐α* (Figure 5A), which even returned to the normal level in the spleen after nano‐windmill treatment. In addition, the nano‐windmill reduced circulating IL‐1β, which was consistent with the in vitro results and our hypothesis that the nano‐windmill could restrain the expression of inflammatory cytokines more strongly than tFNA alone (Figure 5B). Moreover, the nano‐windmill reduced the W/D weight ratio of lung and splenomegaly, identifying its anti‐inflammatory effect (Figure 5C,D). Notably, upon IP injection of 50 mg/kg LPS, the protective effect of the nano‐windmill in mice with sepsis was evaluated by analysing the survival of mice. The results showed that CTL1 inhibition by the nano‐windmill prevented sepsis‐induced death (Figure 5E).

**FIGURE 6:**
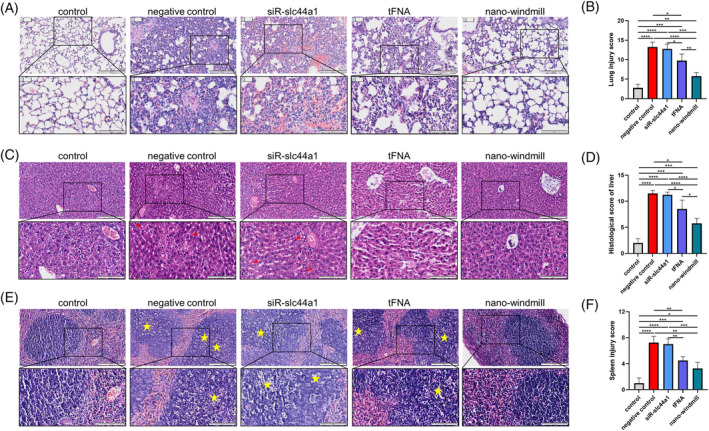
The protective effect of the nano‐windmill on sepsis was observed in H&E staining. (A) The images of lung sections stained with H&E at 10× and 20× magnification. (B) Lung injury scores were estimated and statistically analysed. (C) Representative images of liver sections. The magnification of H&E images is 10× and 20×, respectively. The area indicated by the red arrow represented hepatocyte steatosis. (D) Histological score of the liver was calculated and analysed. (E) Representative images of spleen sections at 10× and 20× magnification. Yellow stars indicated lymphocyte necrosis. (F) Statistical analysis of spleen injury score. Data in (B, D and F) were presented as mean ± SD, *n* = 3. *p < 0.05, **p < 0.01, ***p < 0.001, ****p < 0.0001.

Furthermore, the histological evaluation was performed to validate the anti‐inflammatory effect of the nano‐windmill. We found that the nano‐windmill alleviated LPS‐induced alveolar congestion, thickening of the alveolar wall, neutrophil infiltration, and capillary haemorrhages (Figures 6A and S4), and the lung injury score of mice treated with nano‐windmill was significantly decreased (Figure 6B). In the liver, the nano‐windmill reduced hepatic lobule destruction, hepatocyte edema, cellular infiltration, and haemorrhage induced by LPS (Figure 6C,D). Additionally, white pulps were enlarged in the spleen of mice injected with LPS, coordinated with macroscopic splenomegaly and necrotic lymphocytes, while the nano‐windmill alleviated the inflammatory injury of the spleen and decreased the spleen injury score (Figure 6E,F). These results together demonstrate that the nano‐windmill could alleviate the inflammatory damage of organs induced by sepsis.

### 3.6 The protective effect of nano‐windmill on periodontitis

**FIGURE 7:**
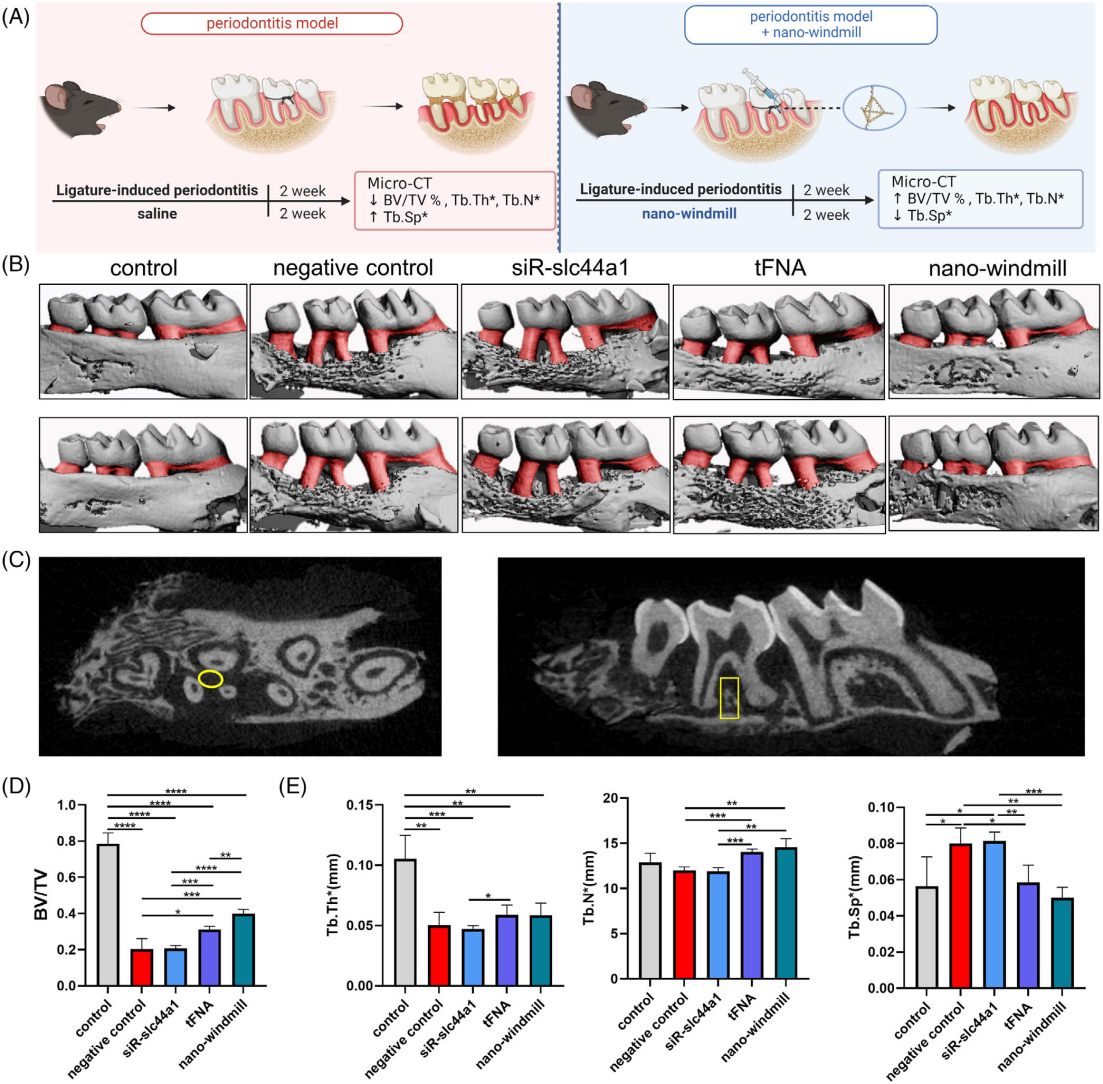
The nano‐windmill inhibited the alveolar bone loss in the mouse periodontitis model. (A) Schematic showing the establishment of mouse periodontitis model and treatment of the nano‐windmill. (B) Three‐dimensional reconstruction images from micro‐CT. (C) The yellow circle and box represented the selected region of interest. (D) Statistical analysis of BV/TV %. (E) Statistical analysis of Tb.N*, Tb.Th* and Tb.Sp*. Data presented as mean ± SD, *n* = 4. *p < 0.05, **p < 0.01, ***p < 0.001, ****p < 0.0001.

NLRP3 inflammasome activation has also been reported to accelerate the progression of chronic inflammatory diseases, such as periodontitis, characterized by exacerbating the absorption of alveolar bone.^44^, ^45^ Thus, the ligature‐induced periodontitis model (Figure 7A) was used to further confirm that the nano‐windmill could exhibit anti‐inflammatory effects in chronic inflammatory disease. Three‐dimensional reconstruction images from micro‐computed tomography (micro‐CT) showed significant alveolar bone loss in the negative control group, identifying the successful establishment of the mouse periodontitis model, and the nano‐windmill decreased the area of dental root exposure, which was marked red (Figure 7B). The region of interest was then selected on the analysis software to analyse the tomography image information obtained by micro‐CT (Figure 7C). Bone volume/tissue volume (BV/TV) was decreased in the negative control group and siR‐slc44a1 group, while it was increased in the nano‐windmill group (Figure 7D). Trabecular thickness (Tb.Th) and trabecular number (Tb.N) of the negative control group were decreased, and the treatment of tFNA and nano windmill could alleviate the reduction of these two indicators, while the trend of trabecular separation/spacing (Tb.Sp) was the opposite (Figure 7E). Collectively, these results indicate that nano‐windmill could exhibit anti‐inflammatory effects in periodontitis by alleviating the alveolar bone loss.

## 4 DISCUSSION

With more and more reports that metabolic reprogramming regulates the phenotype and function of macrophage, researchers believe that anti‐inflammatory therapy can be achieved by targeting macrophage metabolic pathways to regulate macrophage activation.^37^, ^47^, ^48^, ^49^, ^50^, ^51^, ^52^ Increased intracellular phospholipid synthesis depending on choline uptake mainly meets the needs of phagocytosis, organelle biogenesis and secretion after macrophage activation.^9^ In addition, increased choline uptake are commonly observed in inflammatory diseases such as arthritis, periodontitis and cardiovascular diseases.^46^, ^47^, ^48^ Thus, it appears that imparied choline uptake is expected to control inflammation by inhibiting macrophage overactivation.

In this study, reduced choline uptake by decreased *slc44a1* expression was identified as the specific mechanism of tFNA repressing macrophage overactivation. By specific assemble strategy, we successfully developed a novel tFNA‐based nanomedicine for inflammatory disease via amplifying its anti‐inflammatory effect with a much more *slc44a1* knockdown level, which was validated in vivo and in vitro. Our results strongly suggest that the nano‐windmill exerts its anti‐inflammatory role via metabolic reprogramming of macrophages, which should be a novel and promising strategy for anti‐inflammatory therapy, as previous studies discussed.^49^

Moreover, the main advantages of the nano‐windmill are described as follows. First, the nano‐windmill system differentiates from other RNA modifying methods, such as being attached to the sidearm of tFNA or embedded inside tFNA. It breaks into tFNA and siR‐slc44a1 inside the cell without damaging the tetrahedral structure of tFNA, laying the structural foundation for tFNA and siRNA to synergistically decrease the *slc44a1* expression. Second, the nano‐windmill is more biocompatible than other commercial transfection reagents. Third, the nano‐windmill solves the problem that siRNA is difficult to enter cells and easy to degrade. Together, the nano‐windmill can upgrade tFNA to achieve superior anti‐inflammatory effects.

Mitochondrial damage or dysfunction can drive macrophage activation by increasing the production of mitochondrial ROS and cytosolic release of mtDNA.^50^, ^51^ The NLRP3 inflammasome, an innate immune cell sensor, senses mitochondrial dysfunction by detecting the level of ROS and cytosolic mtDNA and promotes the secretion of IL‐1β and IL‐18 in macrophages.^52^, ^53^ Conversely, appropriately enhanced mitophagy is conducive to removing damaged or dysfunctional mitochondria timely, inhibiting abnormal macrophage activation, restricting the secretion of pro‐inflammatory cytokines, and maintaining immune homeostasis.^54^, ^55^ Our results showed that reduced choline uptake by the nano‐windmill decreased the MMP level of macrophages, and then enhanced mitophagy. Due to the accelerated removal of damaged mitochondria by the nano‐windmill, the cytosolic release of ROS and mitochondrial DNA was reduced, thus restraining the excessive activation of NLRP3 inflammasome. This mechanistic finding was validated in mouse models of sepsis and periodontitis, which were NLRP3 inflammasome activation dependent. Overall, although there are few studies about choline metabolism and macrophage activation, our results raise the possibility that targeted inhibition of CTL1 or choline kinase in macrophages may be an effective strategy to inhibit inflammatory responses.

## 5 CONCLUSION

The nano‐windmill developed by a noval tFNA‐based strategy was found to inhibit NLRP3 inflammasome activation by maintaining mitochondrial homeostasis, ultimately repressing macrophage overactivation and alleviating inflammation. Therefore, our results suggested that enhanced targeting of *slc44a1* with the nano‐windmill is a promising therapeutic way for inflammatory diseases.

## AUTHOR CONTRIBUTIONS

**Nanxin Liu, Xiaoxiao Cai and Ping Ji:** Conceptualization and methodology. **Nanxin Liu, Yuke Zhong, Xiaoxiao Pang and Mingzheng Li:** Validation, formal analysis and data curation. **Nanxin Liu, Richard D. Cannon and Li Mei:** Writing and visualization. **Xiaoxiao Cai and Ping Ji:** Project administration. **Nanxin Liu and Ping Ji:** Funding acquisition.

## FUNDING INFORMATION

This study was supported by the National Natural Science Foundation of China (82001058, 82071115, 82220108019), Natural Science Foundation of Chongqing (CSTB2022NSCQ‐MSX0791), and Natural Science Foundation of Sichuan Province (2021YFQ0064).

## CONFLICT OF INTEREST STATEMENT

The authors declare that they have no known competing financial interests or personal relationships that could have appeared to influence the work reported in this paper.

## Supporting information


**Data S1:** Supporting Information.

## Data Availability

The data that support the findings of this study are available on request from the corresponding author upon reasonable request.
