# Supplementary material for: The nano‐windmill exerts superior anti‐inflammatory effects via reducing choline uptake to inhibit macrophage activation
Source: Cell Prolif. 2023 Apr 13;56(10):e13470. doi: 10.1111/cpr.13470 (PMC10542611; doi:10.1111/cpr.13470)
Supplement: Supplementary file 1 — Data S1: Supporting Information. [file CPR-56-e13470-s001.docx]

Supporting Information

**The nano-windmill exerts superior anti-inflammatory effects via reducing choline uptake to inhibit macrophage activation**

*Nanxin Liu^†^, Yuke Zhong^†^, Xiaoxiao Pang, Mingzheng Li, Rechard D. Cannon, Li Mei, Xiaoxiao Cai^*^, Ping Ji^*^*


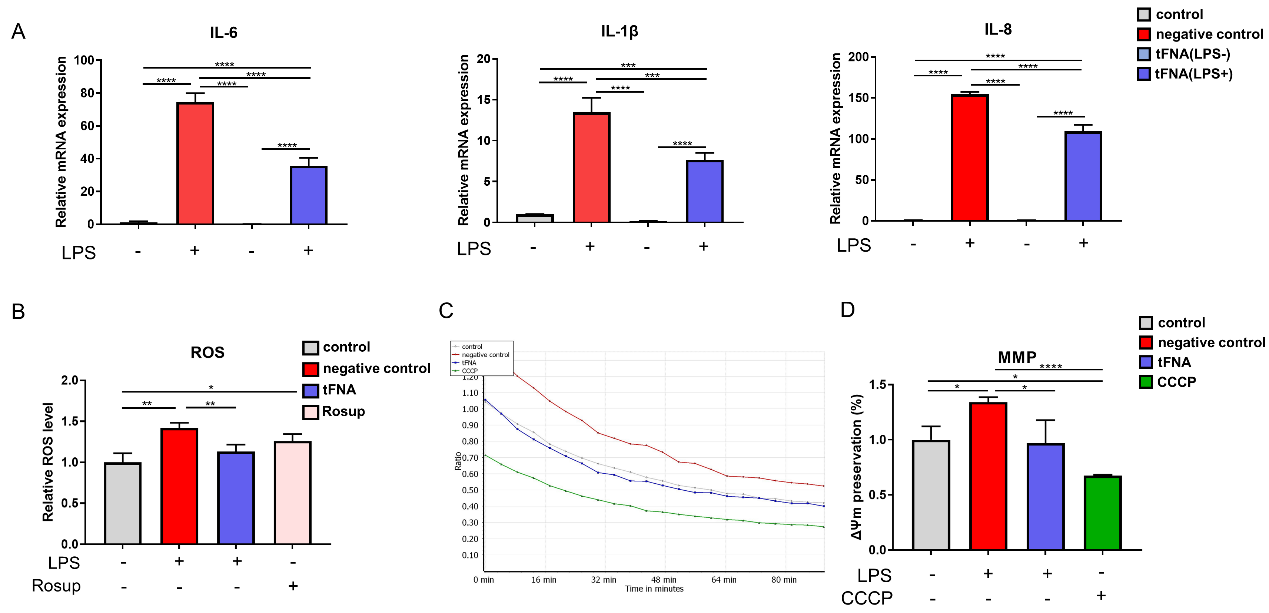
**1.Design, synthesis and characterization of the nano-windmill**

**Figure S1**. tFNA controlled inflammation in macrophages. [A] QPCR analysis of *IL-6*, *IL-1β,* and *IL-8* in PMA-differentiated THP-1 cells treated as indicated for 6 h, n=3. [B] Analysis of ROS level measured by BMG. Rosup was used as the positive control, n=3. [C] Measurement of MMP by BMG. The uncoupler carbonyl cyanide m-chlorophenyl hydrazone (CCCP) was used as a positive control. [D] Statistical analysis of MMP in cycle 5, n=3.


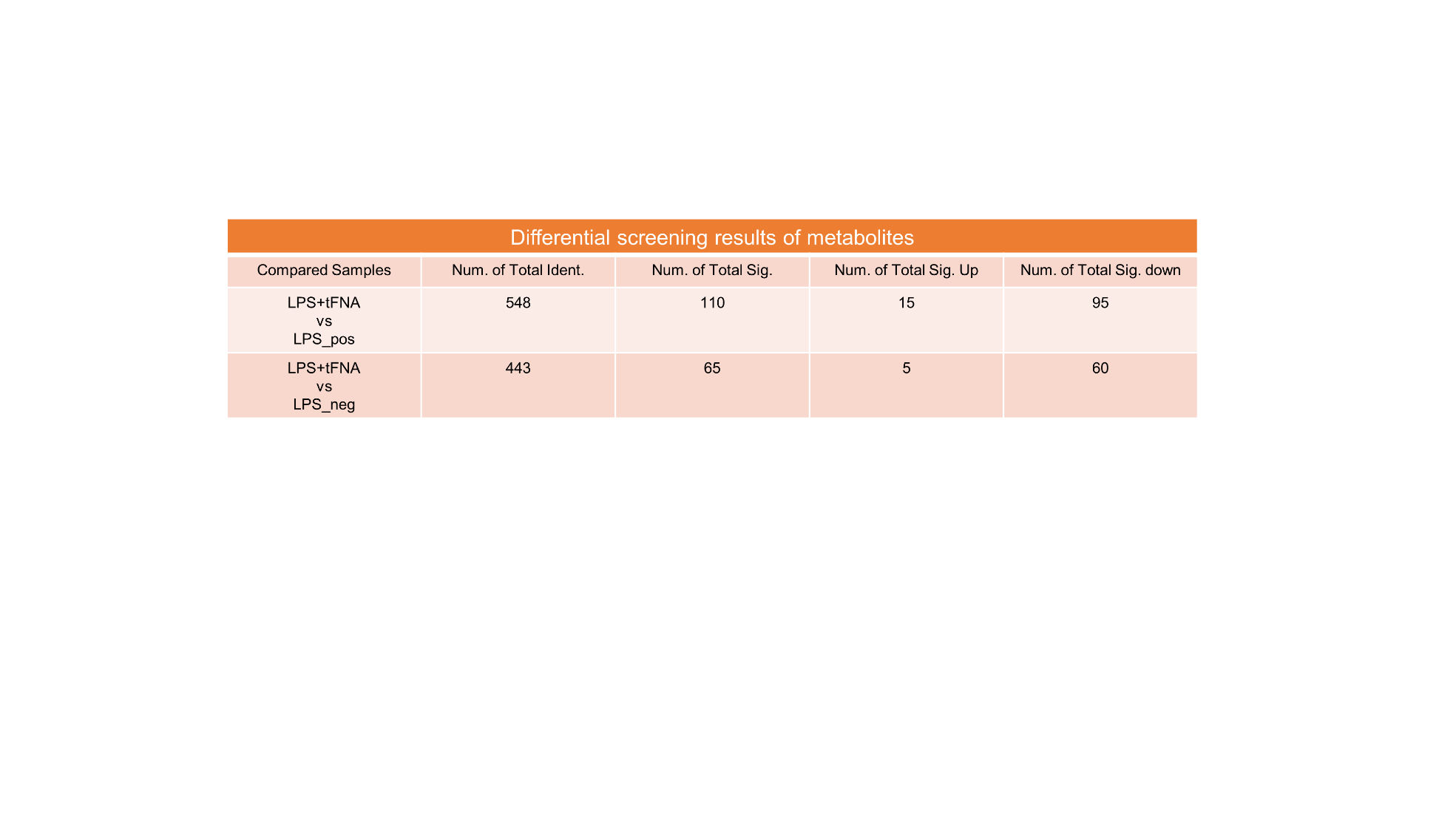
**Table S1**. Differential screening results of metabolites

**
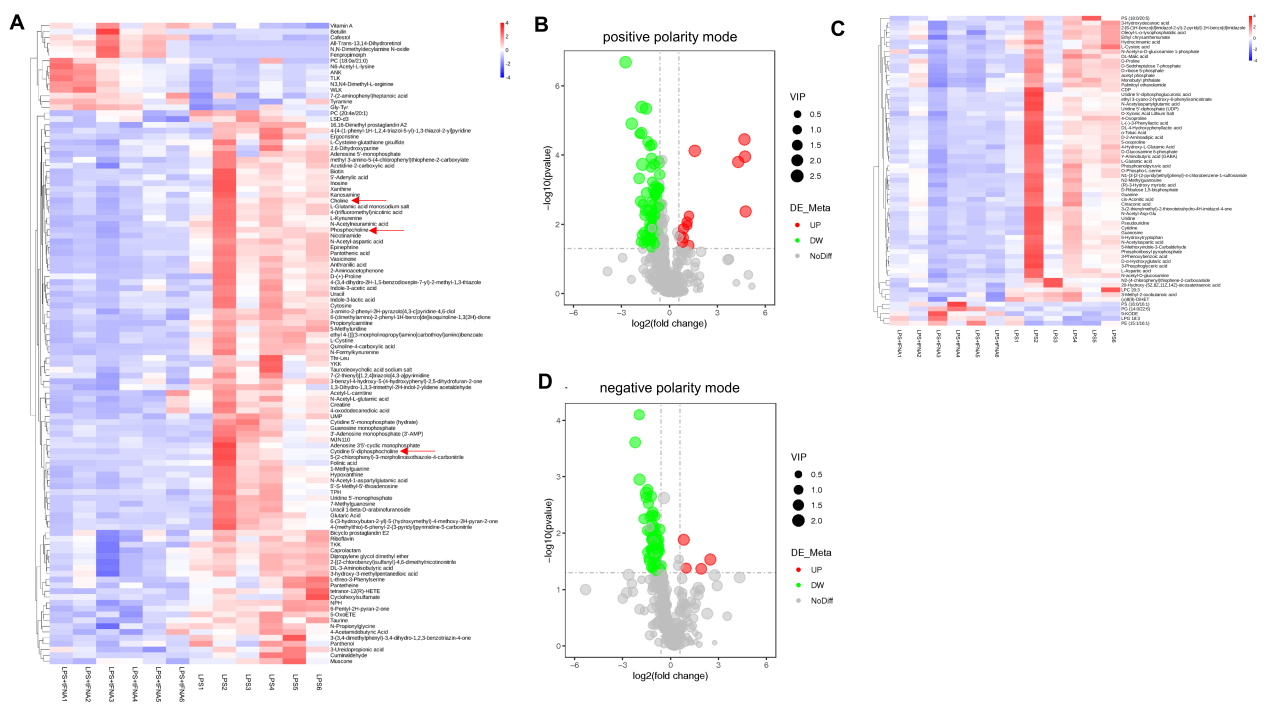
Figure S2**. The differential metabolites of macrophages treated by LPS and tFNA. [A] The heat map of differential metabolites was obtained in the positive polarity mode, n=6. [B,C] Volcano plots showing differences between the LPS (100 ng/ml) group and LPS (100 ng/ml) + tFNA (250 nM) group in number and magnitude of metabolites, n=6. [D] The heat map of differential metabolites in the negative polarity mode, n=6.

**
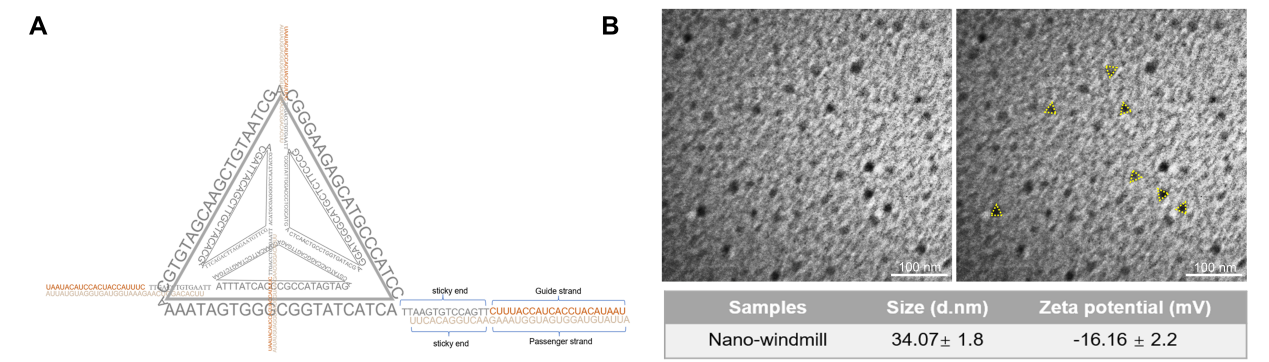
**

**Figure S3**. The composition and characterization of the nano-windmill. [A] The base sequence of nano-windmill including stFNA, siR-slc44a1, and sticky ends. [B] The average size, morphology and Zeta potential were analyzed by TEM and DLS.

**Table S2**. The DNA base sequence of tFNA and stFNA and the RNA base sequence of siR-slc44a1

| Strand | Sequence (5’-3’) |
| --- | --- |
| S1 | ATTTATCACCCGCCATAGTAGACGTATCACCAGGCAGTTGAGACGAACATTCCTAAGTCTGAA |
| S2 | ACATGCGAGGGTCCAATACCGACGATTACAGCTTGCTACACGATTCAGACTTAGGAATGTTCG |
| S3 | ACTACTATGGCGGGTGATAAAACGTGTAGCAAGCTGTAATCGACGGGAAGAGCATGCCCATCC |
| S4 | ACGGTATTGGACCCTCGCATGACTCAACTGCCTGGTGATACGAGGATGGGCATGCTCTTCCCG |
| sS1 | TTGACCTGTGAATTATTTATCACCCGCCATAGTAGACGTATCACCAGGCAGTTGAGACGAACATTCCTAAGTCTGAA |
| sS2 | TTGACCTGTGAATTACATGCGAGGGTCCAATACCGACGATTACAGCTTGCTACACGATTCAGACTTAGGAATGTTCG |
| sS3 | TTGACCTGTGAATTACTACTATGGCGGGTGATAAAACGTGTAGCAAGCTGTAATCGACGGGAAGAGCATGCCCATCC |
| sS4 | TTGACCTGTGAATTACGGTATTGGACCCTCGCATGACTCAACTGCCTGGTGATACGAGGATGGGCATGCTCTTCCCG |
| Guide strand of slc44a1 | UAUCAAGUCCAGGUUGCAUGG |
| Passenger strand of slc44a1 | CCAUGCAACCUGGACUUGAUA |
| Passenger strand of slc44a1 with a sticky end | UUCACAGGUCAACCAUGCAACCUGGACUUGAUA |
| Guide strand of NC | ACGUGACACGUUCGGAGAA |
| Passenger strand of NC with a sticky end | UUCACAGGUCAAUUCUCCGAACGUGUCACGU |

**Figure S4**.
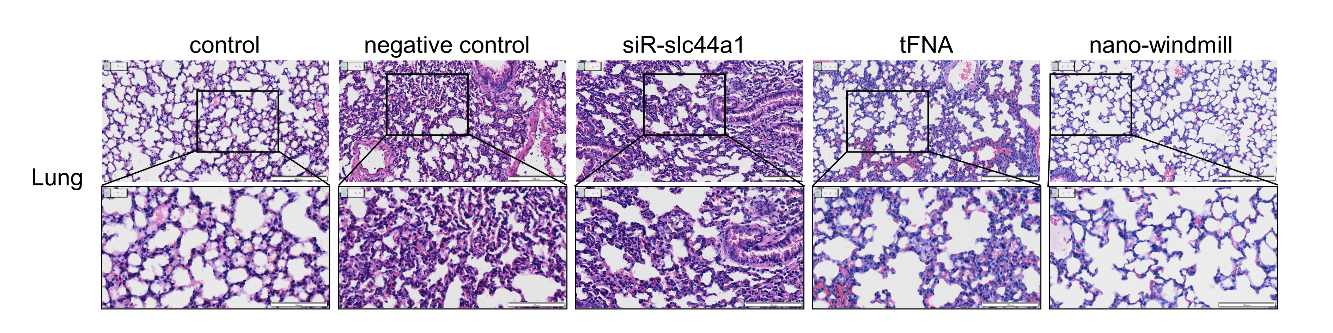
The nano-windmill alleviated lung injury in septic mice.
